# Protein Sequence Classification with Improved Extreme Learning Machine Algorithms

**DOI:** 10.1155/2014/103054

**Published:** 2014-03-30

**Authors:** Jiuwen Cao, Lianglin Xiong

**Affiliations:** ^1^Institute of Information and Control, Hangzhou Dianzi University, Zhejiang 310018, China; ^2^School of Mathematics and Computer Science, Yunnan University of Nationalities, Kunming 650500, China; ^3^School of Mathematics and Statistics, Yunnan University, Kunming 650091, China

## Abstract

Precisely classifying a protein sequence from a large biological protein sequences database plays an important role for developing competitive pharmacological products. Comparing the unseen sequence with all the identified protein sequences and returning the category index with the highest similarity scored protein, conventional methods are usually time-consuming. Therefore, it is urgent and necessary to build an efficient protein sequence classification system. In this paper, we study the performance of protein sequence classification using SLFNs. The recent efficient extreme learning machine (ELM) and its invariants are utilized as the training algorithms. The optimal pruned ELM is first employed for protein sequence classification in this paper. To further enhance the performance, the ensemble based SLFNs structure is constructed where multiple SLFNs with the same number of hidden nodes and the same activation function are used as ensembles. For each ensemble, the same training algorithm is adopted. The final category index is derived using the majority voting method. Two approaches, namely, the basic ELM and the OP-ELM, are adopted for the ensemble based SLFNs. The performance is analyzed and compared with several existing methods using datasets obtained from the Protein Information Resource center. The experimental results show the priority of the proposed algorithms.

## 1. Introduction

**Figure 1:**
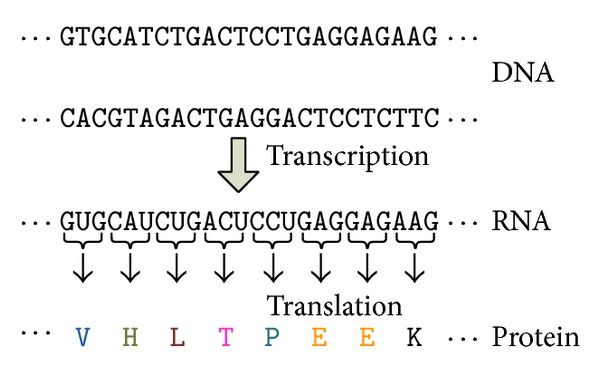
The DNA sequence of a gene encodes the amino acid sequence of a protein.

Protein sequences (also known as polypeptides) are organic compounds made of amino acids arranged in a linear chain and folded into a globular form. The amino acids in a polymer chain are joined together by the peptide bonds between the carboxyl and amino groups of adjacent amino acid residues. The sequence of amino acids in a protein is defined by the sequence of a gene, which is encoded in the genetic code. As shown in Figure 1, a gene is any given segment along the deoxyribonucleic acid (DNA) that encodes instructions which allow a cell to produce a specific product. Typically, a protein such as an enzyme initiates one specific action.

Due to the wide applications in clinical proteomics and protein bioinformatics, protein sequence analyses have been comprehensively studied in recent years, such as the work presented by Barve et al. [1], Chen et al. [2], Cong et al. [3], Machado et al. [4], Carregari et al. [5], and Liu et al. [6]. Protein sequence analysis generally helps to characterize protein sequences in silico and allows the prediction of protein structures and functions. Recent research has shown that the comparative analysis of the protein sequences is more sensitive than directly comparing DNA. Hence, a number of protein sequence databases have been established in the past decades, such as Protein Information Resource (PIR) (http://pir.georgetown.edu/), Protein Data Bank (PDB) (http://www.pdb.org/pdb/home/home.do), and Universal Protein Resource (UniProt) (http://www.uniprot.org/). Hence, it becomes an important and challenging task to efficiently exploit useful information from the large protein sequence dataset for both computer scientists and biologists. As mentioned by Baldi and Brunak [7], protein sequence classification plays an important role in protein sequence analysis on the account of those protein sequence members consisting of a same protein superfamily are evolutionally related and functionally and structurally relevant to each other. Precisely classifying a member protein sequence into a superfamily protein would show the benefit that it only needs to carry out some molecular analysis within a particular superfamily instead of the analysis on all the individual member protein sequences. Generally, two protein sequences are classified into the same category if their feature patterns extracted by sequence alignment algorithms show high homology. Lots of alignment algorithms have been proposed in the past few years to identify the class of the unseen protein sequence based on comparing it with some known protein sequences and calculating their similarities, such as iPro-Class (http://pir.georgetown.edu/), SAM (SAM: Sequence Alignment and Modeling Software System, Baskin Center for Computer Engineering and Science, http://www.cse.ucsc.edu/researchcompbio/), and MEME (MEME: Multiple Expectation Maximization for Motif Elicitation UCSD Computer Science and Engineering, http://meme.sdsc.edu). However, it is a very time-costing work to compare the testing protein sequence with all the existing identified protein sequences, especially when the database is large and the length of the unseen protein sequence is long. Therefore, establishing an efficient and intelligent classification system to exactly label the testing protein sequence in a large database becomes urgent and useful.

A number of methods have been developed for general signal classifications based on the statistical theory in the past decades, such as decision trees, statistical techniques, support vector machine (SVM), and neural networks (NN). Yang et al. [8] employed the word segmentation method for feature extraction on the protein sequence and then utilized the SVM for classification. Beside this, Caragea et al. [9] used the hashing function to reduce the dimension of the protein sequence feature vector and then performed classification with SVM. Alternative to using the SVM method, neural networks are another popular method for protein sequences classification in terms of the following two reasons: (i) as the features of protein sequences are generally distributed in a high dimensional space with complex characteristics, it is usually difficult to find a satisfactory model using the statistical or parameterized approaches, and (ii) neural networks are able to process the raw continuous values fed into the model. A lot of research works based on neural networks for protein sequences classification have been done in the last few years, such as Wang et al. [10, 11] and Wang and Huang [12]. Wang et al. [10] proposed a modular radial basis function (RBF) neural network classifier for the protein sequences with improved classification criteria, and two heuristic rules were presented for the decision-making to enhance the classification reliability. A generalized radial basis function (GRBF) neural network architecture that generates a set of fuzzy classification rules was developed for protein sequences classification by Wang et al. [11]. Most of the previous papers in protein sequence classifications usually chose the gradient based algorithm for neural networks, which are time-consuming in general. Hence, a computationally efficient tuning-free algorithm named extreme learning machine (ELM) for single hidden layer feedforward neural networks (SLFNs), which was recently proposed by Huang et al. [13, 14] and further improved by Huang et al. [15], was applied for protein sequences classification by Wang and Huang [12]. The experimental results given by Wang and Huang [12] have shown that the ELM algorithm learns thousands times faster than the conventional gradient based method (also known as backpropagation (BP), which was developed by Levenberg [16] and Marquardt [17]) with a higher classification rate in protein sequences. To enhance the classification performance and keep the training time in an acceptable level, a self-adaptive evolutionary ELM (SaE-ELM) which utilized the self-adaptive differential evolutionary to update the hidden neuron parameters in the ELM neural network has been presented in Cao et al. [18].

Although the basic ELM and its invariant SaE-ELM have been employed and discussed for protein sequence classification by Wang and Huang [12] and Cao et al. [18], respectively, there are still a lot of rooms for improvements. As presented and discussed by Wang and Huang [12], although ELM learning is much faster than the conventional BP algorithm on the protein sequence dataset, the improvement of classification rate is relatively small. With this objective, we study classification performance of protein sequence based on recent improved ELM algorithms in this paper. The contributions of the paper are threefold. First, the recent robust and generic algorithm named the optimal pruned ELM (OP-ELM) developed by Miche et al. [19] is utilized for protein sequence classification in this paper, where the multiresponse sparse regression (MRSR) technique developed by Simila and Tikka [20] and the leave-one-out (LOO) validation criterion are employed in the OP-ELM for the selection of an appropriate number of hidden neurons. Second, the ensemble based SLFNs network structure is proposed and the majority voting method is adopted to further enhance the protein sequence classification performance. Third, both the basic ELM and the OP-ELM are used as the training algorithms for each ensemble in the new structure. Thus, two algorithms named the voting based ELM (V-ELM) and the voting based OP-ELM (VOP-ELM) are developed for protein sequence classifications. The performance of all the proposed methods is analyzed using the protein sequence database from the Protein Information Resource (PIR) center. Simulations results are compared with recent state-of-art algorithms, such as SVM by Hsu and Lin [21], BP by Haykin [22], Marquardt [17], Levenberg [16], and the original ELM by Huang et al. [13, 14, 23].

Organizations of the rest of the paper are as follows. The feature extraction method for biological protein sequence data used by Wang et al. [10, 11] is reviewed in Section 2. The data description of the protein sequences downloaded from the PIR database is also introduced in this section. In Section 3, the recent ELM and its improved method OP-ELM for protein sequence classification using single hidden layer feedforward neural network are first given. The ensemble based SLFNs structure combined with the majority voting method is then proposed for protein sequence classification. The original ELM and the OP-ELM are used as the learning algorithms for each ensemble. Experimental results and performance comparisons are given in Section 4. Discussions and conclusions are drawn in Section 5.

## 2. Feature Extraction

As described by Wang et al. [10, 11], a protein sequence is generally made from various combinations of 20 amino acids with notations as Σ = {*A*, *C*, *D*, *E*, *F*, *G*, *H*, *I*, *K*, *L*, *M*, *N*, *P*, *Q*, *R*, *S*, *T*, *V*, *W*, *Y*}. For protein sequence classifications, the *n*-gram features with a pair of values (*v*_*m*_, *c*_*m*_) are extracted as the input signals fed to a classifier, where *v*_*m*_ is the feature *m* and *c*_*m*_ is the count of this feature in a protein sequence with *m* = 1,2,…, 20^*n*^. For instance, the 2-gram features from the set Σ are all the combinations as (*AA*, *AC*,…, *AY*, *CA*, *CC*,…, *CY*,…, *YA*, *YC*,…, *YY*). A feature is the number of occurrences of an amino within a protein sequence. Taking a protein sequence *V* *AA* *GT* *VA* *GT* as an example, the 2-gram features can be extracted and presented as {(*VA*, 2), (*AA*, 1), (*AG*, 2), (*GT*, 2), (*TV*, 1)}. Another commonly used information for protein sequence feature extraction is the 6-letter exchange group. That is, the 6 combinations of the letters from the whole set Σ are formed as **A** = {*H*, *R*, *K*}, **B** = {*D*, *E*, *N*, *Q*}, **C** = {*C*}, **D** = {*S*, *T*, *P*, *A*, *G*}, **E** = {*M*, *I*, *L*, *V*}, and **F** = {*F*, *Y*, *W*}. Therefore, using the 6-letter group, the above protein sequence *V* *AA* *GT* *VA* *GT* can be transformed as **E** **D** **D** **D** **D** **E** **D** **D** **D** and its 2-gram features are {(**D** **E**, 1), (**E** **D**, 2), (**D** **D**, 5)}. Similar to the feature extraction done by Wang et al. [10, 11] and Wang and Huang [12], we use **e**_*n*_ and **a**_*n*_ to represent *n*-gram features from the 6-letter group and the 20-letter set, respectively. Each set of *n*-gram features from a protein sequence, that is, **e**_*n*_ and **a**_*n*_, is scaled separately to avoid skew in the counts value using the formula: $\overset{-}{x}=\left(x/\left(l-n+1\right)\right)$, where *x* is the count of the generic gram feature, $\overset{-}{x}$ is the normalized *x*, which is the inputs of the classifiers, **l** is the length of the protein sequence, and *n* is the size of the *n*-gram features.

A 56-dimensional feature vector, extracted from a protein sequence and comprised from *n*-gram features of the 6-letter group represented as **e**_2_ and the 20 letters set represented as **a**_1_, is used as the input information of the classifiers. Two protein sequence datasets with ten-super families (classes) were obtained from the PIR databases and denoted as PIR1 and PIR2, respectively. There are 949 protein sequences samples in PIR1 and 534 protein sequences samples in PIR2. The details of the ten superfamilies to be classified are Cytochrome c (113/17), Cytochrome c6 (45/14), Cytochrome b (73/100), Cytochrome b5 (11/14), Triosephosphate isomerase (14/44), Plastocyanin (42/56), Photosystem II D2 protein (30/45), Ferredoxin (65/33), Globin (548/204), and Cytochrome b6-f complex 4.2K (8/6), where the first digit in the bracket denotes the number of the protein sequences in the PIR1 database and the second digit represents the number of the protein sequences in the PIR2 database, respectively.

## 3. Methodologies

### 3.1. SLFN for Protein Sequence Classification

#### 3.1.1. Model Description of SLFN

For the supervised learning in SLFNs, a dataset with input signal features and their associated class category is generally available to train the network parameters. Assuming that the available dataset is *𝒜* = {(**x**_*i*_,*t*_*i*_)}_*i*=1_^**N**^, where **x**_*i*_, *t*_*i*_, and **N** represent the feature vector of the *i*th protein sequence, its corresponding category index, and the number of protein sequences, respectively; a single hidden layer feedforward neural network (SLFN) with **J** nodes in the hidden layer can be expressed as

(1) $$\begin{array}{c} o_{i}=\sum_{j=1}^{J}w_{j}g\left(a_{j},b_{j},x_{i}\right),\;i=1,2,\ldots ,N, \end{array}$$

where **o**_*i*_ is the output obtained by the SLFN associated with the *i*th input protein sequence, and **a**_*j*_ ∈ *ℛ* ^*d*^ and *b*_*j*_ ∈ *ℛ*  (*j* = 1,2,…, **J**) are parameters of the *j*th hidden node, respectively. The variable **w**_*j*_ ∈ *ℛ* ^*m*^ is the link connecting the *j*th hidden node with the output layer and *g*(·) is the hidden node activation function. With all training samples, (1) can be expressed in the compact form as

(2) $$\begin{array}{c} O=HW, \end{array}$$

where *W* = (**w**_1_, **w**_2_,…, **w**_**J**_) and *O* are the output weight matrix and the network outputs, respectively. The variable *H* denotes the hidden layer output matrix with the entry *H*_*ij*_ = *g*(**a**_*j*_, *b*_*j*_, **x**_*i*_). To perform multiclasses classification, SLFNs generally utilize the One-Against-All (OAA) method to transform the classification application to a multioutput model regression problem. That is, for a *𝒞*-categories classification application, the output label *t*_*i*_ of the protein sequence feature vector **x**_*i*_ is encoded to a *𝒞*-dimensional vector **t**_*i*_ = (*t*_*i*1_,*t*_*i*2_,…,*t*_*i𝒞*_)^*T*^ with *t*_*i ***c**_ ∈ {1, −1}  (**c** = 1,2,…, *𝒞*). If the category index of the protein sequence **x**_*i*_ is **c**, then *t*_*i ***c**_ is set to be 1 while the rest entries in **t**_*i*_ are set to be −1. Hence, the objective of training phase for the SLFN in (1) becomes finding the best network parameters set *S* = {(**a**_*j*_,*b*_*j*_,**w**_*j*_)}_*j*=1,…,**J**_ such that the following error cost function is minimized:

(3) $$\begin{array}{c} \underset{S}{\min}\;E=\underset{S}{\min}||O-T||, \end{array}$$

where *T* = (**t**_1_, **t**_2_,…, **t**_**N**_) is the target output matrix. The most popular algorithm is the gradient descent based method where the network back-forward errors are used to iteratively update the network parameters. However, the slow learning speed and poor learning scalability limit their applications in large datasets and high dimensional signals, such as the protein sequence classification.

#### 3.1.2. ELM

Alternative to iteratively tuning the network parameters, extreme learning machine (ELM), which was recently developed by Huang et al. [13, 14, 23], claims that random hidden node parameters can be utilized for SLFNs and the hidden node parameters may not need to be tuned. It was stated in Huang et al. [14] that a standard SLFN with **N** hidden nodes using random input weights and biases and the infinitely differentiable activation function can exactly learn **N** arbitrary distinct samples. In such case, the system (2) becomes a linear model and the network parameter matrix can be analytically solved by using the least-square method. That is,

(4) $$\begin{array}{c} W=H^{†}T, \end{array}$$

where *H* ^†^ is the Moore-Penrose generalized inverse of the hidden layer output matrix *H* given by Serre [24]. The universal approximation property of the ELM theory by using random input weights and biases is also presented in Huang et al. [14] to support the algorithm.

In the following, a brief summary of the ELM algorithm is given in Algorithm 1.

As illustrated through simulations given by Huang et al. [14, 23, 25], Liang et al. [26], and Zhang et al. [27], utilizing random hidden parameters in the training phase, ELM not only learns thousand times faster than the BP algorithm and its variants but also has a comparable generalization performance as the conventional gradient descent based methods and SVM. The protein sequence classification based on ELM has been first studied by Wang and Huang [12]. The experimental results obtained by Wang and Huang [12] have shown that ELM performs slight better than the BP algorithm where the improvement of the successful testing classification rate obtained by ELM is around 1%. But ELM runs thousand times faster than BP.

#### 3.1.3. OP-ELM

However, using random hidden node parameters and the tuning-free learning framework in ELM may also bring some issues. For example, the parameters may not be the optimal one and redundant nodes may exist. To address these issues and enhance the performance, many invariants of ELM have been developed in the past several years, such as considering the distribution of input dataset done by Cao et al. [28] and utilizing the differential evolutionary method for parameter optimization by Zhu et al. [29] and Cao et al. [30]. One of the recent representative improvement methods named the optimal pruned ELM (OP-ELM) is developed by Miche et al. [19] to prune the related neurons with irrelevant variables. To get rid of unuseful hidden nodes of ELM, OP-ELM combines the multiresponse sparse regression (MRSR) by Simila and Tikka [20] with the leave-one-out (LOO) validation method to rank hidden neurons and to select the actual best number of neurons for the model.

The MRSR technique is described as follows. Suppose **X** = [**x**_1_ … **x**_**m**_] ∈ *ℛ* ^**n**×**m**^ is the regressor and MRSR adds each column of the regressor matrix one by one to the model **Y** ^*l*^ = **X** **β** ^*l*^, where **Y** ^*l*^ = [**y**_1_^*l*^ … **y**_*𝒞*_^*l*^] is the target approximation of the model. At the *l*th step, the weight matrix **β** ^*l*^ has *l* nonzero rows. A new nonzero row and a new column of the regressor matrix are added to the model when increasing the steps. With the MRSR method, the hidden neurons **h**_*i*_ which are the rows of the hidden layer output matrix *H* are ranked. For the details of MRSR, one can refer to the work presented by Simila and Tikka [20].

To select the best number of neurons, the LOO validation method is introduced in OP-ELM. Employing the PREdiction Sum of Squares (PRESS) statistics (presented by Bontempi et al. [31] and Myers [32]), the LOO validation error *ε* ^PRESS^ is calculated as

(5) $$\begin{array}{c} \varepsilon^{\text{PRESS}}=\frac{t_{i}-h_{i}\beta }{1-h_{i}Ph_{i}^{T}}, \end{array}$$

where **P** = (*H* ^*T*^ *H*)^−1^. Then, the appropriate number of neurons for the model is taken by evaluating the LOO error versus the used number of neurons. The OP-ELM can be summarized in three steps as found in Algorithm 2.

### 3.2. Ensemble Based SLFNs for Protein Sequence Classification

**Figure 2:**
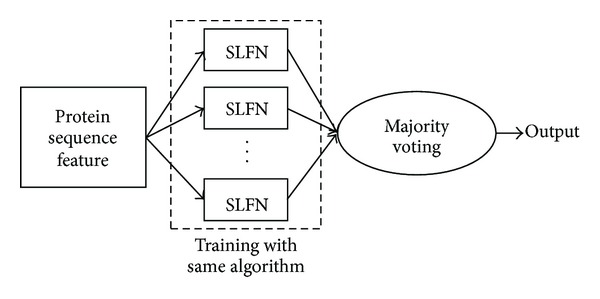
Ensemble structure of SLFNs for protein sequence classification.

For both ELM and OP-ELM, we can find that the hidden node parameters are randomly assigned once and never updated. With a single SLFN training by ELM or OP-ELM on the protein sequence dataset, the misclassification number of samples may be high. To address this issue, we propose an ensemble based structure of SLFNs for protein sequence classification. Since the original ELM enjoys a much fast training speed for SLFN, it is feasible to employ multiple independent SLFNs to predict the category index with the majority voting method. Rather than relying on single realization of random parameters in ELM, employing the ensemble method would be able to reduce the misclassification number. The proposed structure of the ensemble based SLFNs for protein sequence classification is given in Figure 2.

As shown in Figure 2, instead of using a single SLFN, multiple independent SLFNs where each one has the same structure and the same hidden node activation function are used. The protein sequence feature vectors are fed to train each SLFN separately. Then, the final category index of the testing sample is decided by majority voting among all the results obtained by these SLFNs. It is apparent that the gradient descent based methods are not suitable to adopt here as the training algorithm for each SLFN. In general, the training time cost by ensemble based SLFNs is linearly increased with a proportion to the number of independent ensembles. Since the conventional gradient descent methods normally suffer from a long training time for the high dimensional feature vectors and large sample sizes, the training time increases dramatically when employing it for multiple ensembles. Therefore, in this paper, we propose to use the basic ELM and the recent OP-ELM as the training algorithm for each SLFN for protein sequence classification as follows.

#### 3.2.1. The Proposed Ensemble Based ELM

The voting based ELM (V-ELM) developed by Cao et al. [33] is proposed for protein sequence classification in this section. The details are described as follows. We assume that **K** SLFNs are employed as ensembles. In the first stage, **K** sets of hidden parameters {(**a**_*j*_^*k*^,*b*_*j*_^*k*^)_*j*=1,…,**J**_}_*k*=1,…,**K**_ are randomly generated and the corresponding **K** sets of output weight matrix {*W* ^*k*^}_*k*=1,…,**K**_ are obtained with (4) using the protein sequence training dataset *𝒜*. In the second stage, for each trained ELM denoted as *S* ^*k*^ = {(**a**_*j*_^*k*^,*b*_*j*_^*k*^)_*j*=1,…,**J**_,*W* ^*k*^}_*k*=1,…,**K**_, the estimated category index of the testing protein sequence sample **x** ^test^ is obtained. For all **K** ensembles, a *𝒞*-dimensional vector *ℒ*_**K**,**x**^test^_ is used to store all the results where if the category index derived by the *k*th (*k* ∈ [1,…, **K**]) ELM is *ı*, the value of the corresponding entry *ı* in the vector *ℒ*_**K**,**x**^test^_ is increased by one; that is,

(6) $$\begin{array}{c} {ℒ}_{K,x^{\text{test}}}\left(ı\right)={ℒ}_{K,x^{\text{test}}}\left(ı\right)+1. \end{array}$$

In the third stage, the final category index of **x** ^test^ is determined via

(7) $$\begin{array}{c} c^{\text{test}}=\underset{}{\arg}\underset{ı\in [1,\ldots ,𝒞]}{\max}\left\{{ℒ}_{K,x^{\text{test}}}\left(ı\right)\right\}. \end{array}$$

A proposition is also given by Cao et al. [33] to illustrate the superiority of the V-ELM over the original ELM as follows.

**Proposition 1 (see Cao et al. [33])**

Given a standard SLFN with **J** hidden nodes, an output function *g*(·) and a set of training samples {(**x**_*i*_,**t**_*i*_)}_*i*=1_^**N**^ assume that the probability of correctly predicting the testing sample **x** ^*test*^ using ELM under all different possible hidden node parameters **a** and *b* is *𝒫*_*ELM*_(**c** | **x** ^*test*^). If the following inequality holds

(8) $$\begin{array}{c} {𝒫}_{ELM}\left(c\;|\;x^{test}\right)>\max{\left\{{𝒫}_{ELM}\left(ı\;|\;x^{test}\right)\right\}}_{ı\in [1,\ldots ,𝒞],\;ı\neq 𝒞}, \end{array}$$

where *𝒫*_*ELM*_(*ı* | **x** ^*test*^) is the probability that *ELM* classifies **x** ^*test*^ to category *ı* that is different from the class **c**, then, with a sufficiently large independent training number **K**, V-*ELM* is able to correctly classify **x** ^*test*^ with probability one.

A brief summary of the proposed V-ELM for protein sequence classification is described in Algorithm 3.

#### 3.2.2. The Proposed Ensemble Based OP-ELM

Besides using ELM as the training algorithm for each ensemble, we propose the voting based OP-ELM (VOP-ELM) for the protein sequence classification in this section. For each ensemble, the OP-ELM algorithm is incorporated as the training algorithm instead of using the original ELM. Similar to V-ELM, each SLFN ensemble is trained using the OP-ELM method on the protein sequence dataset *𝒜* independently. After training, the category index of each testing sample **x** ^test^ by all the **K** ensembles is obtained. Similar to (6), the *𝒞*-dimensional vector *ℒ*_**K**,**x**^test^_^OP^ is updated via the following equation:

(9) $$\begin{array}{c} {ℒ}_{K,x^{\text{test}}}^{\text{OP}}\left(ı\right)={ℒ}_{K,x^{\text{test}}}^{\text{OP}}\left(ı\right)+1. \end{array}$$

Then, the final category index of the testing protein sequence is obtained by

(10) $$\begin{array}{c} c^{\text{test}}=\underset{}{\arg}\underset{ı\in [1,\ldots ,𝒞]}{\max}\left\{{ℒ}_{K,x^{\text{test}}}^{\text{OP}}\left(ı\right)\right\}. \end{array}$$

A brief summary of the proposed VOP-ELM for protein sequence classification is illustrated in Algorithm 4.

We can find that the proposed VOP-ELM would have a better performance than the V-ELM for each ensemble. Compared with V-ELM, the hidden neurons of each ensemble in VOP-ELM are optimized using the OP-ELM given in Section 3.1. However, the drawback of VOP-ELM is that the cost training time may increase as for each SLFN in VOP-ELM, the MRSR technique, and the LOO validation are performed for hidden neurons selection.

## 4. Experimental Results

In this section, the classification results and discussions on the protein sequence dataset from the Protein Information Resource center are presented. We study the performance in two scenarios. In the first scenario, the PIR1 dataset with 949 samples is fixed as the training dataset while the PIR2 dataset with 534 samples is used as the testing dataset. In the second scenario, the PIR1 and PIR2 datasets are first mixed together and then the training and testing datasets with 949 and 534 samples, respectively, are randomly generated from the mixed dataset. For each protein sequence, a 56-dimensional feature vector is extracted as introduced in Section 2 and hence, the number of nodes in the input layer of the SLFN is 56. The proposed ensemble based VOP-ELM and V-ELM are implemented for the protein sequence classifications. The OP-ELM developed by Miche et al. [19] is also used for comparisons. To compare the classification performance, the original ELM by Huang et al. [14, 23], one of the fastest algorithms of the BP's variants, namely, the Levenberg-Marquardt method by Haykin [22], Levenberg [16], and the SVM by Hsu and Lin [21], is employed for the protein sequence classifications. Three different kernel functions are used as the activation function in the SLFN and SVM, which are the linear kernel function (denoted as **L**), the sigmoid kernel function (denoted as **S**), and the Gaussian kernel function (denoted as **G**), respectively. To maintain the training time under an acceptable level, only **K** = 3 ensembles of SLFNs are used for VOP-ELM. However, for V-ELM, **K** = 7 ensembles of SLFNs are employed due to that the V-ELM utilizes the original ELM as training algorithm and it learns much faster than OP-ELM. For the fairness of comparison, all these simulations are executed in the MATLAB 7.4 environment running on an ordinary PC with 3.0 GHz CPU and 4.G RAM memory. Simulations with SVM are carried out using the compiled C-code SVM package: Libsvm (http://www.csie.ntu.edu.tw/~cjlin/libsvm/) running in the same PC. For SVM, the cost parameter *c* and the kernel parameter *γ* are searched in a grid as proposed by Hsu and Lin [21] and the best combination of these parameters is then obtained in terms of the generalized performance. For all these methods, multiple independent trials of simulation are tested and the average results are reported in the paper. For the BP method, 10 trials are used due to its long training time while, for the rest approaches, 50 trials are utilized. The number of hidden nodes for the BP method is 80. When further increasing the number of the nodes, it usually runs out of memory in our PC, which means the computational complexity of the BP algorithm is very high when processing the protein sequence. All the features of the protein sequence are normalized within the region [−1,1].

### 4.1. Performance on Fixed Training and Testing Datasets

In this section, we fix the protein sequence PIR1 as the training dataset while the protein sequence PIR2 is used as the testing dataset.  Table 1 shows the comparisons of the average successful testing classification rate (Rate) and its standard derivation (Dev) among multiple trials for all the classifiers.  Table 2 gives the corresponding training time cost by all the classifiers and their comparisons.

As highlighted in the boldface in Table 1, the proposed ensemble based VOP-ELM has the highest successful classification rate among all 6 approaches. It can be seen that the proposed VOP-ELM offers an improvement of 7.12% and 1.12%, an improvement of 4.98% and 1.13%, an improvement of 5.89% and 1.41% over the original ELM and the OP-ELM by using the linear kernel **L**, the sigmoid kernel **S**, and the Gaussian kernel **G**, respectively. The V-ELM method performs better than the original ELM and BP in general. The improvement of the classification rate obtained by V-ELM over the original ELM is 2.06%, 2.48%, and 2.66% with the linear kernel, the sigmoid kernel, and the Gaussian kernel, respectively. But the performance of V-ELM is slightly worse than VOP-ELM, OP-ELM, and SVM. It is also worth pointing out that, for all these approaches, using nonlinear kernels (the **S** and **G** functions) generally achieves a higher recognition rate than using the linear kernel. As expected, the original ELM has the fastest training speed. As shown in Table 2, the training phase of the original ELM can be finished less than 1 second (s). It learns ten thousand times faster than BP, thousand times faster than SVM and VOP-ELM (the **S** and **G** kernels), and hundred times faster than OP-ELM (the **S** and **G** kernels). In addition, the training time cost by V-ELM is linearly proportional to the number of ensembles we have used as compared to the training time by the original ELM. With only 7 ensembles, the training phase of V-ELM still can be finished within 1 second, as underlined in Table 1.

**Figure 3:**
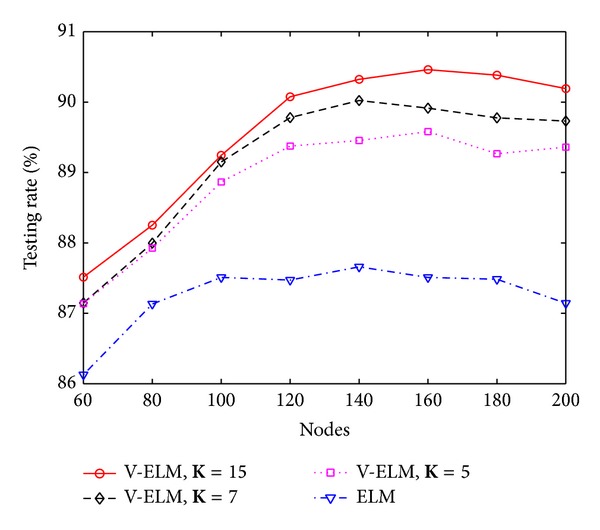
The classification rates of ELM and V-ELM w.s.t. different nodes on fixed training and testing protein sequence datasets.

**Figure 4:**
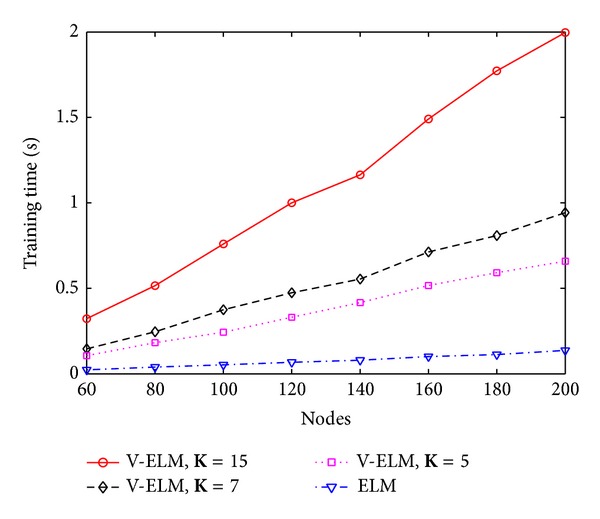
The training time of ELM and V-ELM w.s.t. different nodes on fixed training and testing protein sequence datasets.

In general, a large number of SLFNs used as ensembles usually guarantees a higher classification rate than using a small number of SLFNs as ensembles, but the training time also linearly increases. To illustrate this, three different numbers of ensembles with **K** = 5, **K** = 7, and **K** = 15 are tested on different hidden nodes in each SLFN. The performance is compared with the original ELM. Figures 3 and 4 depict the classification rate and training time of ELM and V-ELM on using the protein sequence PIR1 as training dataset and the PIR2 as the testing dataset. As demonstrated in these two figures, the classification rate increases along with the number of ensembles while the training time also increases gradually. Even though, we can still verify from Figure 4 that V-ELM can finish the training phase within several seconds in general, which makes it acceptable for large sample sizes protein sequence applications. The performance of VOP-ELM with respect to (w.s.t) different numbers of ensembles is not shown here. This is because when further increasing the numbers of ensembles in VOP-ELM to 7 or more, the training time jumps to hundreds or thousands seconds. It will affect its feasibility of applications on large protein datasets.

### 4.2. Performance on Randomly Generated Training and Testing Datasets

In this experiment, the protein sequence datasets PIR1 and PIR2 are first mixed into one file after the feature extraction. Then, for each trial, 939 samples are randomly generated from the whole dataset as the training dataset and the rest samples are assigned to the testing dataset.  Table 3 lists the average successful testing classification rate and the standard derivation of the 6 classifiers among all trials. The training time for the 6 classifiers and their comparisons is shown in Table 4.

From Table 3, it can be seen that the proposed VOP-ELM wins the highest classification rate among all classifiers for all 3 kernels. The VOP-ELM achieves the classification rates of 97.30%, 98.19%, and 98.68% by using the linear, sigmoid, and Gaussian kernels, respectively. The improvements offered by the VOP-ELM method over the original ELM, BP, and OP-ELM are around 2.36%, 2.37%, and 1.35% with the linear kernel, 1.47%, 1.90%, and 1.77% with the sigmoid kernel, and 2.03%, 3.14%, and 1.13% with the Gaussian kernel, respectively. The ensemble based V-ELM performs slightly worse than the VOP-ELM. However, V-ELM is better than the rest methods in general when using the nonlinear kernels. Similar to the experiment in Section 4.1, for the randomly generated training and testing protein sequences, the classifier employing the nonlinear kernel functions (**S** and **G**) generally outperforms using the linear kernel function (**L**).

As shown in boldface in Table 4, ELM is the fastest method and the training phase of the protein sequence dataset takes less than 1 second. However, the conventional BP method takes more than 1 thousand seconds when employing the linear and Gaussian kernels and more than 950 seconds when using the sigmoid kernel. The training time cost by SVM is more than 270 seconds for all the three kernels. Although the original ELM algorithm is employed by the proposed VOP-ELM and OP-ELM, the cost training times jump to dozens of seconds due to utilizing the framework of searching the optimal hidden neurons and multiple ensembles. With only using 7 ensembles in V-ELM, its training time is at the comparable level as the original ELM (as underlined in Table 4). The enhancement of classification rates obtained by V-ELM over the original ELM is 1.94%, 1.03%, and 1.09% for the linear, sigmoid, and Gaussian kernels, respectively.

**Figure 5:**
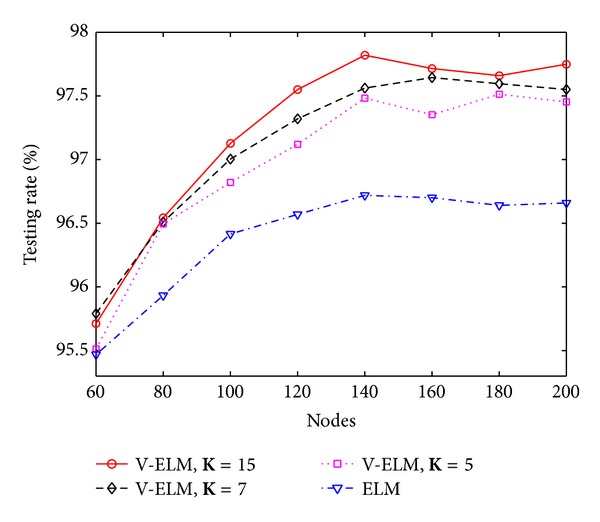
The testing rates of ELM and V-ELM w.s.t. different nodes on randomly generated training and testing protein sequence datasets.

**Figure 6:**
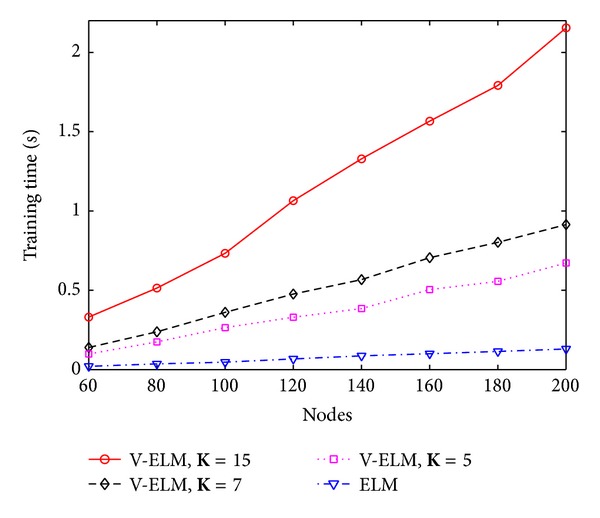
The training time of ELM and V-ELM w.s.t. different nodes on randomly generated training and testing protein sequence datasets.

To further study the performance of V-ELM w.s.t. the number of ensembles, three different numbers of ensembles **K** = 5, **K** = 7, and **K** = 15 are tested on the randomly generated protein sequence datasets. The classification rates and their corresponding training time are compared with the original ELM by using the sigmoid kernel and the results are depicted in Figure 5 and Figure 6, respectively. As illustrated in these two figures, the classification rates obtained by V-ELM gradually increase when the used ensembles are increased from 5 to 15. All the results obtained by V-ELM are better than the original ELM. Although the training times also increase linearly along with the numbers of ensembles, the training phase for V-ELM still can be finished less than 2 seconds, as shown in Figure 6. Similar to Section 4.1, the performance of the proposed VOP-ELM w.s.t. the number of ensembles is not studied in this section because when more than 7 ensembles are used, the training time cost by VOP-ELM will jump to hundreds or thousands seconds. For the large protein sequence dataset, it may not be feasible.

From Tables 1 and 3, it is interesting to see that although the number of samples used from training dataset is the same, the classification rate obtained by using randomly generated protein sequence training dataset is higher than the one by fixing the PIR1 as the training dataset. The reason behind this may be explained as follows. When fixing the PIR1 as the training dataset, some families of the protein sequences become an imbalance problem, such as the families of Cytochrome c, Cytochrome c6, Triosephosphate isomerase, and Globin as shown in Section 2. In such case, the testing samples have a high risk to be misclassified. However, when generating the training and testing dataset from the whole pool, the samples have equal probability to be partitioned into the training dataset and the testing dataset. Hence, the classification model would be trained with a balance dataset, which may result in a high testing rate.

### 4.3. Performance on Different Numbers of Ensembles

In this section, we show the relationship between the number of ensembles used in the proposed algorithms and the classification performance on the protein sequences. The proper number of ensembles which should be adopted in the proposed algorithms is also discussed in the section through the simulations. In the experiments, the number of ensembles is increased from 1 to 51 with an interval 2. The sigmoid kernel is utilized for the experiments as the performance is similar to the one using the other two kernels. The average testing rate for each number of ensembles with 50 independent trials is recorded for both the V-ELM algorithm and the VOP-ELM method. The experiments are running using the PIR1 as the training dataset only. For randomly generated protein sequence dataset, we can find the similar trend of the testing rate and training time on different numbers of ensembles as the one using fixed training dataset. Therefore, the performance on randomly generated training dataset is not repeated here.

**Figure 7:**
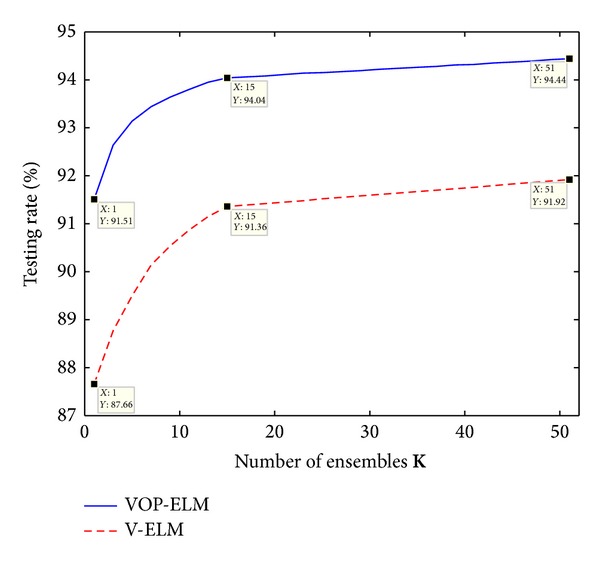
The testing rates of VOP-ELM and V-ELM w.s.t. different numbers of ensembles.

**Figure 8:**
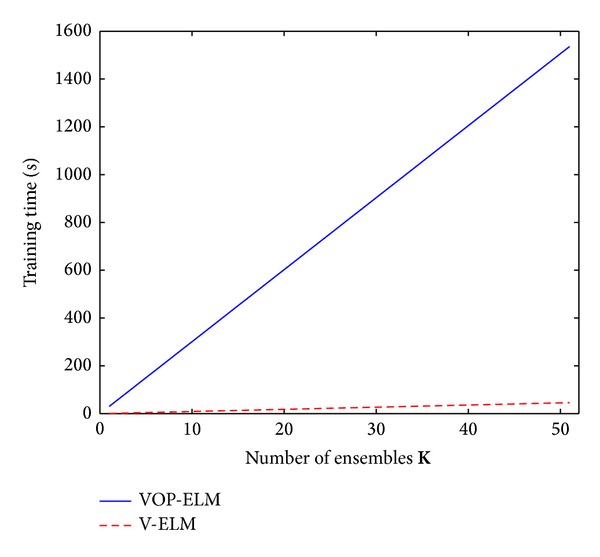
The training time of VOP-ELM and V-ELM w.s.t. different numbers of ensembles.

Figure 7 depicts the testing rate on different numbers of ensembles for the fixed protein sequence training dataset while Figure 8 shows its corresponding training time. As we can find from Figure 7, the testing rate generally increases when the number of ensembles is increasing for both two algorithms. However, the increment is very small when the number of ensemble is larger than 15. It is readily to see that the improvements from using 1 ensemble to using 15 ensembles are around 2.5% and 3.5% for V-ELM and VOP-ELM, respectively. However, when further increasing the number of ensembles from 15 to 51, the improvements of 51 ensembles over 15 ensembles are only around 0.4% and 0.5%, respectively. But the training increases dramatically, especially for the VOP-ELM method. As shown in Figure 8, the training cost by VOP-ELM with 51 ensembles is longer than 1500 seconds. In addition, the training time used for both the two methods is proportional to the training time by the original ELM and OP-ELM, respectively. The proportional rate is related to the number of ensembles. Likewise, we can obtain the similar performance when using randomly generated training protein sequence dataset. Hence, considering both the enhancement of the classification rate and the increment of the training time, we suggest that the best choice of the number ensembles for both V-ELM and VOP-ELM should be less than 15.

## 5. Discussions and Conclusions

As demonstrated by the experimental results in Section 4, the proposed ensemble based VOP-ELM has the highest classification rate and outperforms V-ELM, OP-ELM, SVM, BP, and the original ELM algorithms for the protein sequence recognition. However, employing OP-ELM for each ensemble also increases the training time of the VOP-ELM method, as expected. Even though, VOP-ELM still learns much faster than the conventional BP and the popular SVM.

The original ELM has the fastest training speed of the SLFN model for the protein sequence classification problem among all classifiers. However, the classification performance on protein sequence by ELM is only comparable to the BP method and worse than the proposed ensemble based VOP-ELM and V-ELM, the OP-ELM, and SVM, in general. In addition, using the linear kernel can reduce the training time for some algorithms, such as the OP-ELM and the VOP-ELM algorithms. But the classification rate also reduces when employing the linear kernel as activation function. For the rest of algorithms, the training time used by the linear function is close to the one with the nonlinear kernels, as shown in Tables 2 and 4, respectively. Hence, how to choose the kernel function depends on the requirement of the protein sequence applications.

Employing ELM as the training algorithm for each ensemble, the developed V-ELM outperforms the original ELM in general. The increment of V-ELM compared to ELM is more than 1% using the fixed protein sequence training dataset and is more than 2% using the randomly generated protein sequence training dataset for all the 3 kernels, respectively. Although the training time by V-ELM is longer than the one by ELM, the training phase for the protein sequence dataset still can be finished less than 1 second. Beside ELM, V-ELM can learn many times faster than the rest of compared algorithms. For certain applications, such as developing the online training model for protein sequence analysis, the original ELM and the V-ELM algorithm would be the best choices as researchers normally do a batch model retraining by using the past dataset combined with the new coming samples.

Therefore, as stated above, how to choose the proper classifier for the protein sequence classification depends on the requirements of the application. If only requiring the high recognition rate, the proposed VOP-ELM is the best choice. If the learning speed of the model for the protein sequence is the only concern, the original ELM is the proper classifier. However, if both the high classification rate and the fast training speed are required, the developed V-ELM and the existed OP-ELM should be used.

In conclusion, we have studied the protein sequence classification problem based on SLFNs in this paper. The existed OP-ELM has been first employed as the classifier. Then, the ensemble based structure of SLFNs has been proposed to enhance the performance of protein sequence classification. Two algorithms, namely, V-ELM and VOP-ELM, have been developed for protein sequence classifications by employing the original ELM and the OP-ELM to train each ensemble, respectively. Experimental results on the protein sequence datasets from the Protein Information Resource center demonstrated that VOP-ELM has the highest recognition rate among all compared state-of-art methods while the V-ELM outperforms the original ELM and BP but maintains the training speed as comparable as the original ELM. Moreover, to obtain a reasonable testing rate with an acceptable training time for the ensemble based algorithms, we have shown by simulations that the proper number of ensemble should be chosen less than 15.

## Figures and Tables

**Algorithm 1:**
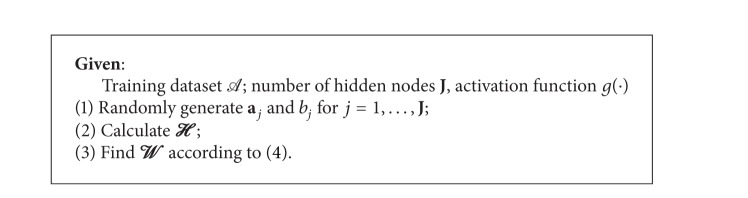
ELM [14, 23].

**Algorithm 2:**
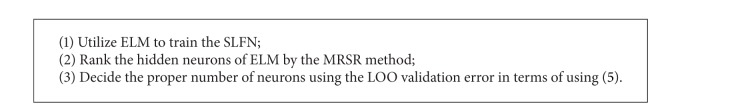
OP-ELM [19].

**Algorithm 3:**
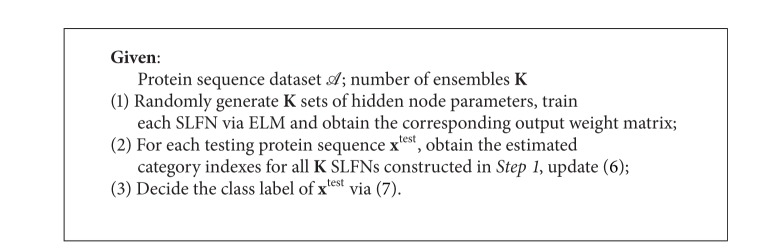
V-ELM.

**Algorithm 4:**
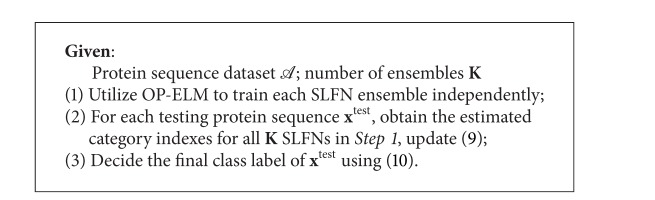
VOP-ELM.

**Table 1:** Successful testing classification rate and standard derivation (rate (%) ± Dev (%)) comparisons of different classifiers: fixed protein sequence training dataset (pir1) and testing dataset (pir2), where **L**, **S**, and **G** stand for the linear kernel, the sigmoid kernel, and the Gaussian kernel, respectively.

Methods	**L**	**S**	**G**
VOP-ELM	**92.51** ± 0	**92.64** ± 1.6	**93.69** ± 0.89
V-ELM	87.45 ± 0.01	90.14 ± 0.71	90.46 ± 0.69
OP-ELM	91.39 ± 0.01	91.51 ± 1.0	92.28 ± 0.70
SVM	90.63 ± 0.01	90.63 ± 0.01	90.63 ± 0.01
BP	85.77 ± 0.42	88.39 ± 1.69	88.77 ± 1.35
ELM	85.39 ± 0	87.66 ± 1.2	87.80 ± 1.4

**Table 2:** Training time comparisons of different classifiers: fixed protein sequence training dataset (pir1) and testing dataset (pir2), where **L**, **S**, and **G** stand for the linear kernel, the sigmoid kernel, and the Gaussian kernel, respectively.

Methods	** L **		** S **		** G **	
	Training time (s)	Speedup	Training time (s)	Speedup	Training time (s)	Speedup
VOP-ELM	5.5685	105.4	86.80	13.37	95.22	13.93
V-ELM	*0.4590 *	1279.3	*0.9020 *	1286.14	*0.8680 *	1528.34
OP-ELM	1.8588	315.90	30.11	38.53	30.45	43.57
SVM	236.16	2.486	236.67	4.90	243.10	5.46
BP	587.20	1	1160.1	1	1326.6	1
ELM	**0.0612**	9594.8	**0.0799**	14519	**0.0792**	16750

**Table 3:** Successful testing classification rate and standard derivation (rate (%) ± Dev (%)) comparisons of different classifiers: randomly generated training and testing datasets from the mixed protein sequences, where **L**, **S**, and **G** stand for the linear kernel, the sigmoid kernel, and the Gaussian kernel, respectively.

Methods	**L**	**S**	**G**
VOP-ELM	**97.30** ± 0.73	**98.19** ± 0.70	**98.68** ± 0.71
V-ELM	96.91 ± 0.71	97.75 ± 0.64	97.74 ± 0.59
OP-ELM	95.95 ± 0.29	96.42 ± 0.45	97.55 ± 0.55
SVM	97.17 ± 0.49	97.28 ± 0.63	97.33 ± 0.66
BP	94.93 ± 0.95	96.29 ± 0.85	95.54 ± 0.91
ELM	94.94 ± 0.75	96.72 ± 0.85	96.65 ± 0.63

**Table 4:** Training time comparisons of different classifiers: randomly generated training and testing datasets from the mixed protein sequences, where **L**, **S**, and **G** stand for the linear kernel, the sigmoid kernel, and the Gaussian kernel, respectively.

Methods	**L**		**S**		**G**	
	Training time (s)	Speedup	Training time (s)	Speedup	Training time (s)	Speedup
VOP-ELM	5.2799	266.44	83.55	11.38	92.00	12.43
V-ELM	*0.6326 *	2223.83	*0.6917 *	1374.51	*0.9282 *	1232.17
OP-ELM	1.8627	755.24	30.93	30.74	32.59	35.09
SVM	278.35	5.05	284.30	3.34	272.41	4.2
BP	1406.8	1	950.75	1	1143.70	1
ELM	**0.0746**	18858	**0.0861**	11042	**0.0983**	11635
